# Climbing with adhesion: from bioinspiration to biounderstanding

**DOI:** 10.1098/rsfs.2015.0015

**Published:** 2015-08-06

**Authors:** Mark R. Cutkosky

**Affiliations:** Department of Mechanical Engineering, Stanford University, Stanford, CA 94305, USA

**Keywords:** bioinspiration, adhesion, gecko climbing, robotics, biology

## Abstract

Bioinspiration is an increasingly popular design paradigm, especially as robots venture out of the laboratory and into the world. Animals are adept at coping with the variability that the world imposes. With advances in scientific tools for understanding biological structures in detail, we are increasingly able to identify design features that account for animals' robust performance. In parallel, advances in fabrication methods and materials are allowing us to engineer artificial structures with similar properties. The resulting robots become useful platforms for testing hypotheses about which principles are most important. Taking gecko-inspired climbing as an example, we show that the process of extracting principles from animals and adapting them to robots provides insights for both robotics and biology.

## 1. Origins and recent growth of bioinspired robotics

The more we learn about the structure and function of plants and animals, the better able we are to extract design principles to guide our own creations. Although people have undoubtedly been inspired by Nature for as long as they have been designing artefacts, some of the first efforts to study Nature systematically and use the resulting insights to design better machines are found in documents from the Renaissance. Artists and philosophers, such as Leonardo da Vinci and Leon Battista Alberti [1], were inspired by what they observed in Nature, arguing that natural proportions and structures should be reflected in art, architecture and engineering.

Leonardo da Vinci clearly devoted considerable time to studying animals such as fish and birds in detail and to dissecting animal and human cadavers to understand their structure and function. In manuscripts, such as his codex on the flight of birds [2], we find direct evidence of bioinspiration, with drawings of birds in flight and bird anatomy juxtaposed with designs for bird-inspired machines.

More generally, from the Renaissance, we find two key reciprocal ideas articulated concerning bioinspired design:

— humans and animals can be thought of as marvellous, complex machines with levers, ropes, conduits, etc.
— the machines that we design are in a sense embodiments of ourselves, with limbs, tendons, etc.

The second of these ideas was more apt during the Renaissance, when machines were mostly human-scaled and human- or animal-powered, than today. Nonetheless, one can find bioinspiration cited in a wide range of current technologies from micromechanical systems [3,4] to composite materials [5].

Moreover, although bioinspiration has clearly informed the design of machines for a long time, there has been a dramatic recent increase in the number of publications that invoke bioinspiration. A quick survey in Google Scholar shows that the number of publications mentioning ‘bioinspired’ or ‘biomimetic’ principles has grown steadily with a little over 12 000 articles from 1990–1999, over 100 000 from 2000–2009 and another 78 000 articles since then. There are also new scholarly journals such as *Bioinspiration & Biomimetics* (IOP Science) devoted entirely to bioinspired advancements.

What is responsible for the recent growth in bioinspired systems and especially in bioinspired robotics? In the case of robotics, one motivation may be that robots are starting to move out of the predictable and structured workplaces, such as the factory floor, and into the world at large. Hence, they need to start displaying some of the robustness that animals do when faced with challenging circumstances. One could argue that the very idea of a robot is inherently bioinspired: engineers seek to create autonomous or partially autonomous entities that can move, sense, take decisions and interact robustly with the world around them, as animals do.

Returning to the opening sentence of this section, a second important reason for the growth in bioinspired robotics may be that we have recently developed tools that allow us to understand in microscopic detail how biological organisms are constructed and how they function. At the same time, engineers have also developed new tools and fabrication processes that allow them to create analogues to the structures and systems found in Nature. The development of gecko-inspired adhesives and climbing robots provides us with an example of these intertwined developments and will be used in the following sections to explore a progression from bioinspiration to biounderstanding, with a mixture of biological and engineering perspectives on adhesion and climbing.

## 2. Gecko-inspired directional adhesion

The woodpecker … can run up and down a tree in any way, even with the head downwards, like the gecko-lizard.

Aristotle, *The History of Animals*^1^ (350 BCE)

Although people have been interested in how geckos stick from at least the time of Aristotle, a true understanding of their adhesive system awaited the availability of scanning electron microscopes and extremely sensitive two-axis microelectromechanical force sensors to view and measure in detail the attractive forces produced by setal stalks as they are brought into contact with surfaces. Scanning electron microscopic images revealed the fine branching of gecko setae and spatulae, which allow conformation to surfaces at microscopic scales [6]. Subsequent investigations of the gecko's hierarchical system of spatulae, setae, lamellae and branching tendons in the toes followed, with comparisons to the generally simpler setal structures found in other lizards and insects [7–9]. These other creatures also exploit adhesion [10–12], but the gecko stands out for its size, weight, agility and the magnitude of the adhesive forces that it can produce.

The attribution of adhesion to van der Waals forces awaited testing with two-axis micromechanical force sensors under a variety of conditions [13,14] and paved the way for numerous efforts to create synthetic dry adhesives [15,16].

**Figure 1.**
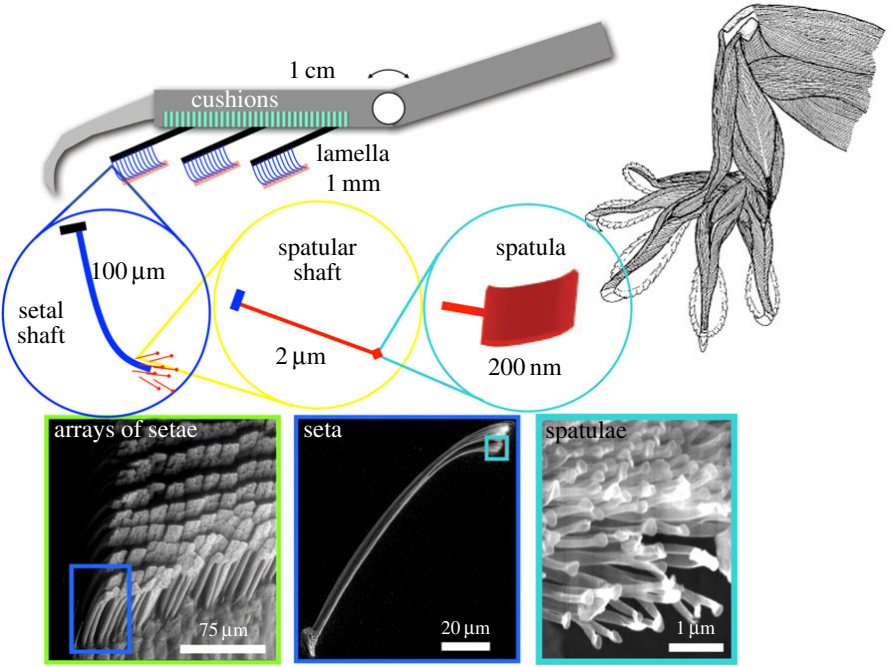
The gecko's hierarchical adhesive system spans a wide range of length scales from centimetres to nanometres. (Reproduced with permission from K. Autumn, Lewis & Clark College.)

From a functional perspective, particularly useful properties of the gecko's adhesive apparatus include [17,18]:

— Conformation: dry adhesion uses van der Waals forces, which only work over molecular distances; hence, a dry adhesive system must conform intimately to a surface to produce useful levels of adhesion. The gecko's hierarchical system of lamellae, setal stalks and spatular tips (figure 1) conforms to both rough and smooth surfaces, producing enough adhesion that the gecko can easily hang from a single toe.
— Directional adhesion: the gecko's adhesion is also directional and controllable. The adhesive system sticks only when pulled from the palm towards the tips of the toes (as in climbing a wall); otherwise, it is not sticky. Moreover, the amount of adhesion varies with the applied tangential force. As seen in §2.1, reproducing this proportionality has been instrumental for climbing with a synthetic adhesive.
— Low effort of attachment and detachment: the gecko runs up and down walls at speeds of the order of a metre per second, taking many steps per second. A low effort of attachment and detachment prevents it from wasting energy with each step.
— High cycle rate: given the number of steps the gecko takes per second, the adhesive must attach and release quite rapidly. Although current climbing robots take only a couple of steps per second, the speed of synthetic gecko-inspired adhesives is useful, as noted in §4.1, for other applications such as small perching air vehicles.
— Self-cleaning: the gecko's feet stay clean even on dusty surfaces [19]. Dirt particles are relatively large compared with the terminal spatulae of the gecko's adhesive system and are more strongly attracted to most wall surfaces than they are to the gecko; hence, the gecko can shed dirt particles when running over most surfaces (with a few exceptions such as white dry-erase boards).

Today, over 100 papers have been published on synthetic and natural gecko-inspired adhesives [16]. There are now several types of gecko-inspired synthetic adhesives that are suitable for various applications including climbing robots, grasping surfaces in manufacturing processes, and even grasping objects such as solar panels and fuel tanks in space [20,21]. The last of these applications is particularly compelling, because dry adhesives are one of the few technologies that will work in a vacuum, at very low temperatures, on non-magnetic materials, and with very low attachment and detachment forces. A few synthetic dry adhesives have even demonstrated levels of adhesion that, for small areas and under controlled conditions, considerably exceed those of the gecko. However, no synthetic adhesive fully captures the desirable properties of the gecko system for climbing. Perhaps for this reason, although there are many publications on dry adhesives, the number of gecko-inspired climbing robots remains small.

### 2.1. A controllable synthetic adhesive

**Figure 2.**
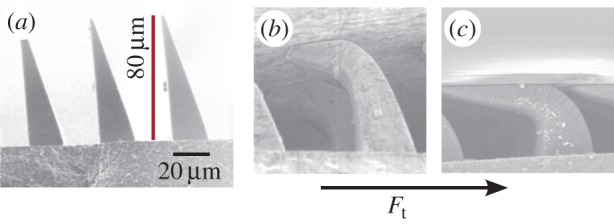
(*a*) Microwedges, like the gecko's adhesive, present a very small area when first brought into contact with a surface. Applying a small (*b*) and subsequently a large (*c*) shear load causes them to bend over, creating a much larger contact area with adhesion. When the shear force is relaxed, they revert to the condition in (*a*).

The directional property of the gecko's dry adhesive has inspired some anisotropic synthetic adhesives [22–27]. In particular, one synthetic adhesive used for the Stickybot climbing robot [28], and other applications, consists of an array of ‘microwedges’ of silicone rubber (figure 2). The wedges have a sharp triangular cross section, approximately 20 µm wide at the base and 80 µm tall. The adhesive is fabricated by casting liquid silicone rubber into a mould which can be fabricated using either lithographic [26] or micromachining [29] techniques.

In the unloaded state (figure 2*a*), the sharp wedge tips present very little contact area as they are brought into contact with a surface; hence, they are not sticky. However, when a gentle (*b*) and finally a large (*c*) shear force, *F*_t_, is applied, they bend over, creating an increasing contact area so that van der Waals forces can produce adhesion. Thus, they represent a greatly simplified analogue to the gecko's setal stalks and spatulae, which also present a small contact area when unloaded, but flatten out for a much larger contact area when pulled in the preferred direction. Although the microwedges have a much lower maximum adhesive stress than gecko setae, they are adequate for climbing robots and other applications.

## 3. Bioinspired design process for climbing with adhesion

**Figure 3.**
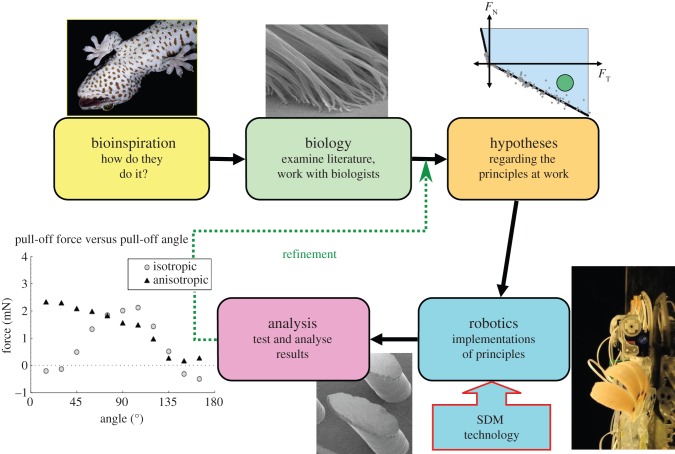
A bioinspired design process for a climbing robot. Insights arise from hypotheses about how complex biological systems can be simplified while preserving important functional attributes. Multi-material prototyping processes (e.g. SDM) make it possible to produce bioinspired robots that embody some of these principles. Testing invariably leads to refinement of the hypotheses.

In 2006, a team of roboticists and biologists from Stanford, Berkeley, Lewis & Clark College, the University of Pennsylvania, Carnegie Mellon and Boston Dynamics, Inc., were engaged in a project on bioinspired climbing robots. The team's process of adapting biological insights into engineering solutions is depicted in figure 3. It begins with an examination of various animals that, in this case, can climb smooth vertical surfaces with agility. Possible exemplars include geckos, lizards, spiders and insects. Among these animals, insects stand out for their ability to run up surfaces using tiny spines on their legs and geckos stand out for their ability to run up both rough and smooth surfaces using adhesion.

The next step of the bioinspired design process involves creating hypotheses about principles that underlie the animals' success. Because we cannot exactly reproduce complex biological structures, we attempt to identify the most important effects, so that we can incorporate them into simplified approximations of what we observe in Nature. We then fabricate robotic mechanisms that embody those principles and test them. In the present case, we use a rapid prototyping process called shape deposition manufacturing (SDM) that allows us to combine hard and soft polymers with embedded fibres and other components for creating bioinspired structures [30].

It is at this stage that robotics can provide useful information for biologists as well as engineers, because it is much easier to conduct comprehensive tests on robots than on animals. We then analyse the results and invariably have to refine our hypotheses and robotic implementations, and so the cycle repeats.

### 3.1. Exploiting controllable adhesion

Investigations of the gecko's adhesive system [13,31] have elucidated the directional nature of its adhesion. For much of the time, a gecko running over a horizontal surface is not sticky; it sticks only when contact forces pull from the palm out towards the toes, as when climbing up a wall or hanging from a ceiling. Indeed, to run head-first down a wall, a gecko must reverse its hind feet to maintain adhesion. The mechanism behind the gecko's directional adhesion is highlighted in the scanning electron microscopic image of loaded setal stalks in figure 3—when pulled in the direction shown, the tips flatten against the surface and provide a relatively large contact area, so that van der Waals forces can produce the desired adhesion. This is the property that the microwedges in figure 2 are attempting to approximate.

**Figure 4.**
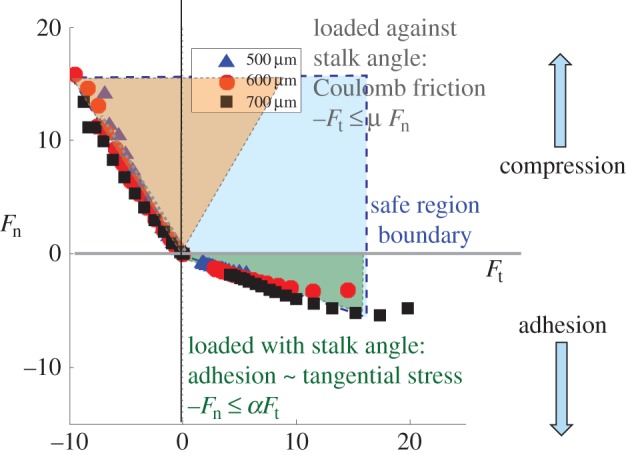
A limit curve in force space. When the normal force is positive and the shear force is negative (from toes towards palm), Coulomb friction pertains; when the tangential force is positive, adhesion is available in proportion to the shear load. (Adapted from [28,32].)

For additional insights, it is useful to recast the observations regarding the gecko's directional adhesive structures in terms of robot force and motion planning. For robot control, it is useful to think of constraints and regions of allowable forces in a multi-dimensional force space. The objective is to plan force trajectories for the robot foot, so that contact forces remain in a safe region. This view of the gecko adhesive forces was first articulated in [32] and is summarized in figure 4, which shows limiting normal and tangential forces for three different levels of preload, corresponding to 500, 600 and 700 µm of elastic compression, respectively. Increasing the preload produces a small increase in the available adhesion. However, a more interesting result is that the adhesion varies almost linearly with tangential force.

When the normal force, *F*_n_, in figure 4 is positive, the foot is pressing into the wall and Coulomb friction pertains: |*F*_t_| ≤ *μF*_n_. However, when a positive shear force is applied, pulling from the palm towards the tips of the toes, some adhesion is available. Moreover, the magnitude of the adhesion is in proportion to the shear force, up to a limit: −*F*_n_ ≤ *αF*_t_ for 0 ≤ *F*_t_ ≤ *F*_t__max_, where *α* is a constant of proportionality, somewhat like *μ* for Coulomb friction. This proportionality makes the adhesion controllable: the gecko can adjust the amount of adhesion that it exerts at any instant by controlling the shear force that it applies with its feet. When taking a step up a wall, it applies a large shear force for maximum adhesion. When ready to detach its foot at the end of a step, it relaxes the shear force, bringing the combined normal and shear force towards the origin of the plot and allowing it to detach its foot with almost no detachment force.

**Figure 5.**
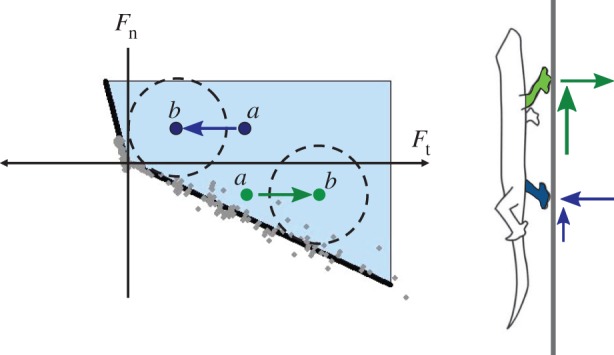
If the weight of the robot is evenly distributed (*a*) between the upper and lower limbs, the front limb has a small margin of safety against detaching from the surface. Controlling the upper limb to carry a larger percentage of the load (*b*) provides a greater overall margin of safety.

The proportional nature of the adhesion also leads to some possibly non-intuitive results. Figure 5 shows a small gecko or robot climbing a vertical wall using a diagonal gait with one upper and one lower foot in contact at each step. Because the centre of mass is located a small distance away from the wall surface, the upper foot, shown in green, must produce adhesion (*F*_n_ < 0) to keep the gecko from falling backward off the wall. The blue lower limb, in contrast, is pressed gently into the wall (*F*_n_ > 0). If we plot the corresponding forces with respect to the adhesion limits, it is clear that the green dot corresponding to the upper limb, initially at position (*a*) in force space, is closer to the edge of the safe region than the blue dot associated with the lower limb. This situation matches our intuition that the upper limb is more likely to fail and may suggest a control approach that tends to ‘favour’ the upper limb by loading it gently and supporting most of the weight with the lower limb. But, this is precisely the wrong strategy! Instead, the gecko or robot should pull harder with its front limbs, so that it has more adhesion with which to work. The result is shown by moving the forces from (*a*) to (*b*) in the figure, so that both feet have an equal safety margin with respect to the limits of adhesion and sliding.

The safe region also makes clear the force trajectory that a gecko-inspired robot should use for climbing smoothly and efficiently. As the robot first brings its foot into contact with the wall, the forces start at the origin. It then starts to apply a shear load parallel to the wall, applying more force to the upper limbs, so that they have adhesion with which to work. To detach its foot, it should relax the tangential force, moving back to the origin where there is no adhesion, and, therefore, no effort to detach from the surface. In practice, adopting this loading and unloading strategy was essential for getting the Stickybot robot to climb smoothly and reliably [4].

**Figure 6.**
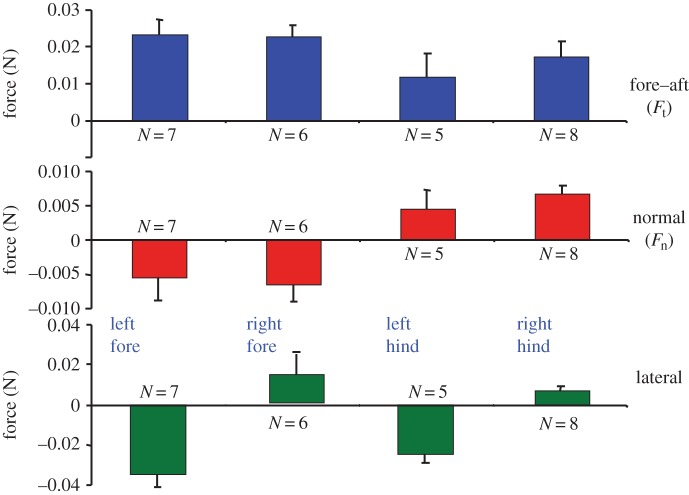
Gecko force data when climbing a vertical wall. (Adapted from Autumn *et al.* [31] with permission.)

The insight afforded by figure 5 also results in a testable hypothesis: if geckos have directional and controllable adhesion, do they also pull harder with their front limbs? As we see in figure 6, this is, indeed, the case. The fore–aft (i.e. tangential) forces that geckos apply with their forelimbs are slightly larger than those from their hind limbs (in contrast to human climbers, who push upward mainly with their legs). In addition, as expected, the normal forces are negative for the forelimbs and positive for the hindlimbs. Finally, the lateral forces pull symmetrically inward towards the spinal axis of the gecko.

## 4. Extensions to perching and human climbing

### 4.1. Dynamic adhesive loading

As noted in §2, the gecko's adhesive system is fast, which allows it to take many steps per second. Although climbing robots are comparatively slow, there are other applications that can take advantage of the potential speed of dry adhesives.

Being lightweight, fast, controllable and easy to attach and detach recommends gecko-inspired adhesives for use with perching microair vehicles (MAVs), allowing them to land on windows and smooth walls and ceilings. MAVs have become widely available in recent years, with applications ranging from aerial photography to environmental monitoring and surveillance [33]. However, a limitation of MAVs, particularly those with a mass of less than 1 kg and using batteries for power, is that they can typically fly for 20 min or less before running low on power. Perching allows them to remain in place for hours, providing a stable, quiet platform for observation.

When perching using directional adhesives, the most important load forces are dynamic, occurring as the MAV hits the wall and rebounds shortly after making contact. Hence, the adhesives must resist dynamic forces in any direction.

**Figure 7.**
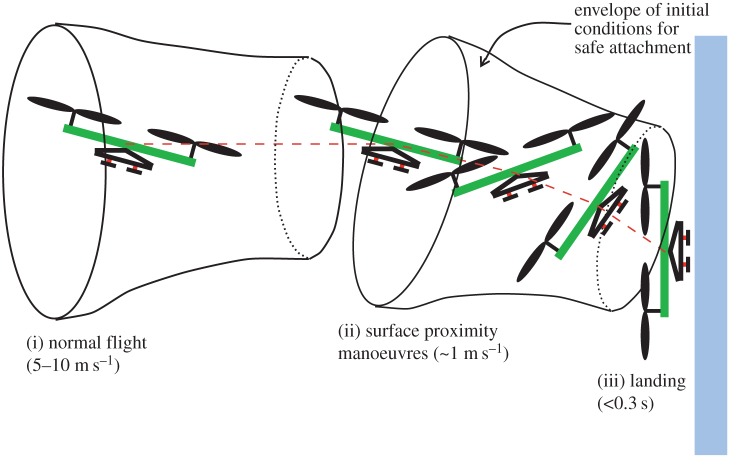
Landing sequence for a small air vehicle: each stage in the sequence can be viewed as a ‘funnel’ in state space. The goal of the manoeuvre at each stage is to get the vehicle within the mouth of the next funnel for a safe landing.

The basic sequence for a perching air vehicle is shown in figure 7. The vehicle normally flies at speeds of up to 10 m s^−1^ and pitches upward to reduce its speed to 1–2 m s^−1^ for landing. This is still rather fast, but is desirable because it makes the MAV much less vulnerable to air currents than a vehicle hovering adjacent to a wall. However, the challenge presented by this dynamic landing is to absorb the kinetic energy of the vehicle, using it to load the adhesives, so that it can latch onto the surface without bouncing off. The entire landing sequence can be viewed as a series of ‘funnels’ in state space; the goal at each stage is to manoeuvre the vehicle so that its position, orientation and velocity put it within the mouth of the funnel corresponding to the next stage.

**Figure 8.**
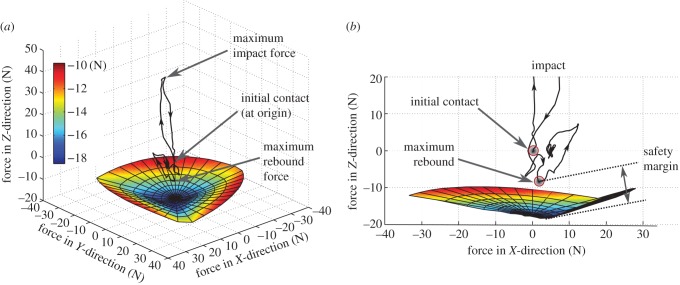
(*a*) Typical landing force trajectory plotted with respect to allowable contact forces for an adhesive gripper with three pads. (*b*) Detail of forces projected in normal/tangential plane showing the safety factor for maximum rebound force. (Adapted from [34].)

As in the case of a climbing robot or gecko, the adhesion limit in force space provides insights into motion planning and mechanism design. In this case, we compare dynamic landing forces to a three-dimensional space of allowable forces in the normal, tangential and lateral directions. Figure 8 shows the forces associated with a typical quadrotor landing on a flat inverted surface. At first contact, the force quickly increases from zero to a maximum impact force. Subsequently, the contact force becomes negative as the quadrotor rebounds. The landing mechanism is designed to withstand such forces with some safety margin.

Here again, we can draw inspiration and potentially useful design principles from animals, including insects, birds, bats and even gliding geckos that move from tree to tree in the rainforest, steering with their limbs and tails and attaching themselves as they land, tail-downward, on tree trunks [35].

In particular, some of the tendon loading and preloading mechanisms used by insects are applicable to perching with MAVs. A challenge in adapting gecko-inspired dry adhesives to perching is that they have a time constant, not captured in the quasi-static loading data from figures 4 and 5. If the normal component of the contact force ramps up too quickly (in less than a few milliseconds), the microscopic structures will not have had time to bend over (as in figure 2*c*) and produce adhesion. Additional design trade-offs include: varying the amount of energy absorbed during compression of the gripper mechanism; changing the amount of damping (which dissipates energy, but increases the initial contact force at high speeds); and varying the properties of the rebound spring to mitigate the peak negative normal force, without producing excessive oscillation [36].

**Figure 9.**
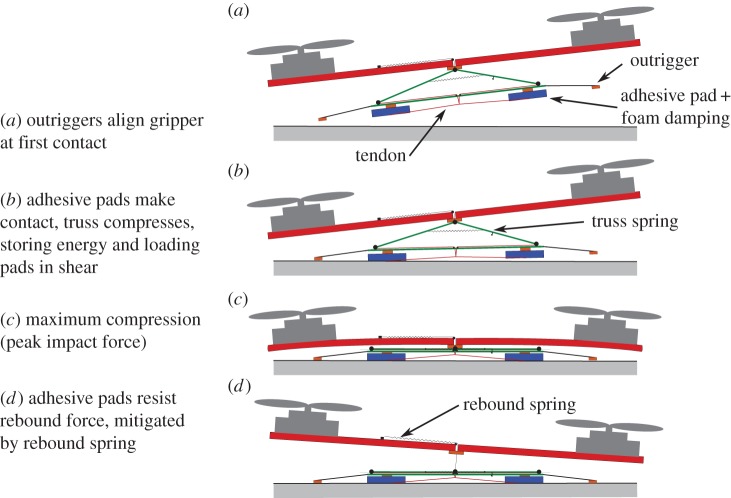
Landing mechanism that absorbs the kinetic energy of an incoming micro air vehicle and uses it to load a pair of adhesive tiles for perching on a smooth surface. (Adapted from [20].)

A solution to the rate dependence of adhesion is to preload the wedges in shear using a mechanism such as that depicted in figure 9. The illustrated gripper has two tiles, each surfaced with an array of microwedges. As it is brought into contact with a surface, a triangular truss linkage at the centre collapses, tensioning the load tendons attached to the adhesive tiles. This happens while the MAV is still in the final stages of pressing against the wall, and preloads the adhesives primarily in shear. When the subsequent maximum rebound force occurs, the tiles are already firmly attached to the surface and can prevent the air vehicle from bouncing off. A separate, nonlinear, rebound spring absorbs the shock of the first bounce, which helps to prevent the gripper from being overloaded.

### 4.2. Scaling to large loads

Although the gecko is an exemplar for using controllable adhesion to climb smooth surfaces rapidly and efficiently, no gecko is particularly large. Even the largest geckos have a mass of well under a kilogram. Nonetheless, we can draw inspiration from the tendon mechanism of the gecko when trying to scale adhesion to larger areas and loads, as might be needed for human climbing or for attaching to large objects in space.

**Figure 10.**
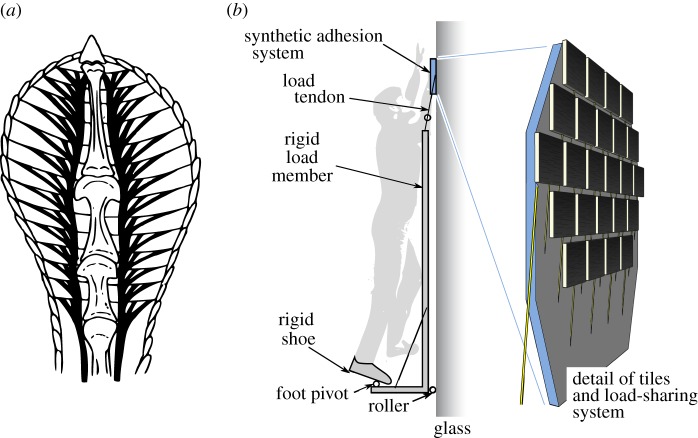
(*a*) Branched tendon structure in gecko toe; (*b*) array of tiles, tendons and nonlinear springs for human climbing. (Adapted from [37].)

Figure 10*a* shows the branching tendon structure of the gecko toe that helps to recruit the entire adhesive area of the toe when a load is applied to the gecko's leg. In addition, the tokay gecko (*Gekko gecko*) has a fluid-filled sinus cavity in the toe that helps to achieve uniform loading as it places its toe against a surface. However, the tendon structure by itself cannot prevent a catastrophic peeling failure when large loads are applied. The problem is that some region of the total adhesive area will inevitably—perhaps as a result of surface irregularities or non-uniformity in the loading system—experience an increased local stress as loads are applied to the system. It will fail and immediately impose an additional load on its immediate neighbours. They also fail, and thus the failure propagates rapidly across the entire system, like an avalanche. As a consequence, prior efforts to use synthetic adhesives for areas of a few centimetres and larger have, in practice, faced rapidly diminishing maximum adhesive pressures. Even the gecko suffers from this problem—indeed, the gecko's adhesive system cannot practically be scaled to human size without changes in the design.

A solution to this problem is to prevent premature local failures by ensuring an absolutely uniform load distribution, despite small geometrical variations. Mechanisms for dividing a load are in the category of a differential—like the differential that splits engine torque between the inner and outer wheels of a car as it is rounding a corner. Such mechanisms have been known for many years and include examples such as the historic whiffle tree that allows a team of oxen to share the load when pulling a heavy cart. Differential mechanisms are effective, but tend to become heavy and complex when dividing a load among many sites.

An alternative, demonstrated in [37], is to use an array of degressive springs with the behaviour that, as they are stretched, they reach a plateau for which modest changes in the stretched length result in almost no change in the elastic force. Using this approach, an array of 24 postage stamp-sized tiles and load tendons is configured into a paddle that supports a human climber (figure 10).

## 5. From bioinspiration to biounderstanding

We have used the gecko and its adhesive system as an example of biological inspiration for synthetic adhesives, with applications to climbing robots, perching air vehicles and even human climbing. As noted in the Introduction, there are many other examples of bioinspired materials and systems and entire conferences and academic journals now devoted to advancements stemming from bioinspiration.

As Leonardo da Vinci understood very well, bioinspiration is clearly good for design. But what good is it for science? To paraphrase a co-worker, ‘It's great that you were inspired by biology, but what hypothesis are you confirming? Your machine is inspired by biology. What knowledge have we gained?’

The contribution of bioinspired design lies in adapting and simplifying what we observe in Nature. Whenever we examine biological systems in detail, we discover a daunting level of complexity. Organisms achieve this complexity by growing and differentiating cell by cell. The cost of complexity in Nature is accordingly small. As an illustration, consider that the gecko not only produces the remarkable hierarchical adhesive structure composed of lamellae, setal stalks and spatular tips shown in figure 1, but this structure is also inert, like hair or fingernails. It regrows its entire skin and adhesive structure anew each month as it moults.

In contrast, when making synthetic gecko adhesives, we use bulk manufacturing processes such as lithographic patterning and micromachining. As we progress from microscopic to macroscopic features, we typically need to employ entirely different processes and machines. New manufacturing and prototyping processes such as micromachining and shape deposition manufacturing expand our repertoire of materials, dimensional scales and geometries, but do not overcome the limitation that each additional level of hierarchy and complexity is costly.

Fortunately, we do not need to reproduce the complexity found in biological systems. Whatever we are designing, however versatile, is far more single purpose than any structure or system in Nature. Our robots may need to operate in multiple different environments, but they do not have to eat or procreate. Hence, we should be careful about drawing single-purpose conclusions regarding any particular structures or behaviours that we observe.

Moreover, natural selection is not engineering optimization—if a new feature is advantageous, it will be preserved and perhaps extended; if it is disadvantageous, it will be deselected; if neither particularly advantageous nor disadvantageous, it is likely to be carried along as baggage from generation to generation. As a simple example, those who suggest that the optimal number of digits for dexterous manipulation is five should ask why we also have five toes. Indeed, many vertebrates, geckos included, also have five digits. Are five really optimal for all of these applications?

The inherent differences between man-made and natural solutions force us to generate simplifying hypotheses about what we think is truly important for accomplishing a certain, functional behaviour. Figure 3 highlights the iterative nature of this process: as we test our ideas with robotic creations, we confirm or reject our hypotheses, providing insights for both designers and biologists. Biounderstanding is the critical element to any functional solution. Da Vinci produced elegant, avian-inspired machines through simple observation of the natural world but it took 400 more years of insight for the Wright brothers to first take flight.

## Endnote

1 http://classics.mit.edu/Aristotle/history_anim.9.ix.html
